# Anesthesia outside the Operating Room in a Patient with Mitochondrial Disease

**DOI:** 10.1155/2020/7902820

**Published:** 2020-05-16

**Authors:** Alejandra Fadrique-Fuentes, Beatriz Martínez-Rafael, Rodrigo Poves-Álvarez, Estefanía Gómez-Pesquera

**Affiliations:** Department of Anesthesiology and Resuscitation, Hospital Clínico Universitario de Valladolid, Valladolid, Spain

## Abstract

Mitochondrial dysfunction comprehends a wide range of genetic disorders. These patients' precarious metabolic balance makes its management difficult. Furthermore, the same systems affected by mitochondrial disease can be altered by many of the frequently used anesthetic agents. Each patient has to be evaluated individually according to their comorbidities and anesthetic requirements.

## 1. Introduction

Mitochondrial diseases represent a broad group of different genetic and clinical disorders, affecting 1 per 5,000 live births.

Mitochondria are the eukaryotic cell's main source of energy. It obtains ATP through oxidative phosphorylation and cellular respiration. The mitochondria are the only organelle with its own DNA, maternally inherited. Any inherited or acquired DNA mutation can affect ATP production as well as fatty acid oxidation.

The decrease or lack in ATP production severely affects high-energy-requiring systems [1]: the central nervous system, cardiovascular system, digestive tract, and muscular tissue. Encephalopathies, ataxias, myopathies and cardiomyopathies, and hepatic and gastrointestinal diseases are frequent. As cells are not able to obtain ATP, the risk of lactic acidosis and hypoglycemia is increased by the rise of anaerobic glycolysis and cellular ketogenesis [2].

Because of their comorbidities and potential complications, diagnostic and therapeutic procedures in patients with mitochondrial disorders frequently require general anesthesia or sedation. It is important to analyse every patient individually, as well as prevent possible metabolic decompensation [1].

### 1.1. Case Presentation

An auditory-evoked potential test (AEP) was performed on a 21-year-old woman with a history of mitochondrial disease and severe progressive hearing loss. She carried an acquired mutation in gene *G* 7896a (subunit COX II, complex IV) of the mitochondrial respiratory chain. On physical examination, she presented a Tanner III constitutional delay (26 kg and 135 cm, below the 3th percentile for weight and height). She also suffered from hypertrophic myocardiopathy with preserved biventricular function and moderate mental retardation because of the metabolic encephalopathy. Her daily medications included propranolol and vitamin *E*.

The auditory-evoked potential test was administered in a specific room (covered by a Faraday cage) outside the surgical area. It was necessary to accommodate the room with a mechanical ventilator, drug trolley, and a wide choice of ventilation and vascular devices.

As the patient was uncooperative after hospital admission, parental presence was requested in order to decrease her anxiety.

She presented the following baseline vitals after monitoring: oximetry, 98% room air SpO2; capnography, EtCO2 34; basal glycaemia, 110 mg/dl; electrocardiography, sinus rhythm 100 bpm; and blood pressure, 110/60 mmHg. Depth of anesthesia was also monitored (PSI (Sedline®)).

Inhalational induction was performed with a decreasing concentration of sevoflurane (8% to 2%) in (10 l/min) 50% oxygen in air and administered with a 1l Mapleson circuit. A 20-gauge intravenous (IV) catheter was placed in a dorsal hand vein. Anesthesia was maintained by target-controlled infusion (TCI) with 2.5 *µ*g/ml of propofol. The administration of sevoflurane was stopped after the induction.

The patient maintained hemodynamic stability all over the procedure. Increasing doses of propofol were required (plasma concentration goal of 3 *µ*g/ml) to obtain PSI values between 45 and 55. Despite high hypnotic doses, spontaneous ventilation was maintained. Oxygen was administrated via nasal cannula (4 l/min). Ranitidine (25 mg iv) and ondansetron (2.6 mg iv) were also used. 250 ml of 0.3% glucosaline were infused during the 90-minute procedure.

After the AEP test, the patient was admitted to post-anesthesia care unit (PACU). She was transferred under mild IV sedation (single propofol bolus of 1.5 mg.kg-1) in the left lateral decubitus position, with Venturi mask (FiO2 of 0.5).

In PACU, she presented the next arterial blood analysis results: pH, 7.3; PaO2, 102 mmHg; PaCO2, 47. The patient was discharged to the ward one hour after well-tolerated early feeding, without incidents.

## 2. Discussion

Deeper level sedation was needed in order to decrease the patient's anxiety during the procedure. The anesthetic approach had to be careful because of significant risk of metabolic decompensation and growth delay [1, 3]. General anesthesia with airway manipulation was reserved if required.

### 2.1. Preanesthetic Considerations

The procedure was performed directly after hospital admission seeking to lower the patient's metabolic and psychological stress (increased by her cognitive dysfunction, deafness, and communication difficulties). Parental presence was required while monitoring and face mask collocation. Strong family attachment and the presence of a known element had an important anxiolytic power [4].

Benzodiazepine sedation can be useful in mitochondrial disease by suppressing hypermetabolism secondary to stress. Although benzodiazepines are considered safe in these patients [5], they inhibit complexes I, II, and III of the electron transport chain in a dose-dependent way [2]. In this case, their use was avoided because of the increased risk of side effects (overdose and respiratory complications).

It is very important to reduce preoperative fasting times and to maintain normal glycaemia and pH values [1, 2]. If needed, not in this case, glucose must be replaced (by glucose solution or 5% dextrose) as well as bicarbonate in case of metabolic acidosis.

### 2.2. Anesthetic Considerations

In mitochondrial disorders, anesthesia must be as short as possible. Only the indispensable dose of drugs should be administered. Hypothermia, nausea, and vomiting increase the metabolism and the risk of acidosis. Active patient warming measures would be required in case of important heat loss procedures. It is appropriate to use antiemetic prophylaxis [1].

### 2.3. Anesthetic Agent

The choice of anesthetic agents remains a controversial issue. To a greater or lesser extent, every general anesthetic drug depresses mitochondrial function. A review of 111 cases of general anesthesia in adult patients with mitochondrial disease was published in 2016 [6], where the use of intravenous rather than inhalation anesthesia was recommended. However, one year later, in 2017, A. Smith et al. [1] concluded that there was no consistent relationship between the anesthetic agent used and the subsequent perioperative complications. There is consensus about the need to slowly titrate the anesthetic dose [2], as these patients present lower anesthetic requirements.

Volatile anesthetics affect oxidative phosphorylation by inhibiting complex I NADH dehydrogenase function [6]. They depress ventilatory response to CO2 (isoflurane and desflurane) and muscle tone (sevoflurane) as well as increase the incidence of postoperative nausea and vomiting.

Despite of it not being frequent, the use of inhalation agents increases the risk of malignant hyperthermia in patients with mitochondrial myopathy [6]. Moreover, the lack of gas evacuation systems outside the operating room limits their use.

However, their main benefit lies in its rapid respiratory induction and elimination, which is not affected by mitochondrial function [2]. Volatile anesthetics are widely used in sedation outside the operating room because of the hemodynamic and respiratory stability they allow. They are considered safe drugs even in patients with myopathy [7]. Sevoflurane is the most appropriate inhalation agent in mitochondrial disease.

Propofol infusion outside the operating room involves less technical difficulties. It does not increase the risk of malignant hyperthermia, has an antiemetic effect, and increases convulsive threshold [9].

Propofol use is essentially limited by apnea episodes that may require airway manipulation as well as hospital admission in 0.18% of patients, according to Srinivasan et al.'s study [8]. Although propofol usually enables a mild induction and awakening, its hepatic metabolism can be affected by mitochondrial disease. There is a very low risk of propofol-related infusion syndrome [6] if used at low doses for a short time. It is important to note that propofol inhibits complexes I and IV of the electron transport chain [2].

Owing to the lack of a clear indication, sedation with propofol infusion was considered the safest anesthesia to maintain spontaneous breathing in this case. Sevoflurane was used at the beginning of the procedure to enable the canalization of a peripheral cannula and a quick start of the anesthesia. It was done according to Yemen and McClain publication [7], which recommends changing to intravenous anesthesia after inhalation induction in order to decrease infrequent but severe volatile agent adverse effects in patients with myopathy.

### 2.4. Fluid Therapy

Hypovolemia and hypotension increase the risk of acidosis in mitochondrial disease. Lactate solutions must be avoided as buffer's metabolism also decreases pH [1, 2]. Should dextrose be used, its concentration must be below 5%. In this particular case, 250 ml of 0.3 glucose-saline was infused to replace the blood stream.

### 2.5. Postanesthetic Considerations

The patient was admitted to PACU where an arterial blood gas analysis was performed. Parental presence was required until PACU discharge, and early feeding was started as soon as possible, seeking to decrease metabolic and psychological stress [4].

Patients with mitochondrial disease require a close monitoring. Analysis is required to replace blood deficits during and after most of the procedures [1, 2].
